# Internet addiction among adolescents in Macau and mainland China: prevalence, demographics and quality of life

**DOI:** 10.1038/s41598-020-73023-1

**Published:** 2020-10-01

**Authors:** Dan-Dan Xu, Ka-In Lok, Huan-Zhong Liu, Xiao-Lan Cao, Feng-Rong An, Brian J. Hall, Gabor S. Ungvari, Si-Man Lei, Yu-Tao Xiang

**Affiliations:** 1 Unit of Psychiatry, Faculty of Health Sciences, University of Macau, 3/F, Building E12, Avenida da Universidade, Macao, SAR China; 2 Department of Biology, Faculty of Sciences, Harbin University, Harbin, China; 3 Department of Psychiatry, Chaohu Hospital, Anhui Medical University, Hefei, Anhui China; 4 Anhui Psychiatric Center, Anhui Medical University, Hefei, Anhui China; 5 Shenzhen Children’s Hospital, Shenzhen, China; 6 The National Clinical Research Center for Mental Disorders & Beijing Key Laboratory of Mental Disorders, Beijing Anding Hospital & The Advanced Innovation Center for Human Brain Protection, Capital Medical University, Beijing, China; 7 New York University (Shanghai), Shanghai, China; 8 Division of Psychiatry, School of Medicine, University of Western Australia, Perth, Australia; 9 University of Notre Dame Australia, Fremantle, Australia; 10 Faculty of Education, University of Macau, Macao, SAR China; 11 Center for Cognition and Brain Sciences, Institute of Advanced Studies in Humanities and Social Sciences, University of Macau, Macao, SAR China

**Keywords:** Addiction, Public health, Quality of life

## Abstract

Internet addiction (IA) is common among adolescents and significantly determined by sociocultural and economic factors. The aim of this study was to compare the prevalence of IA among adolescents between Macau and mainland China and also examine its association with quality of life. A total of 2892 secondary school students were included. Standardized instruments were used to measure IA, depressive symptoms and quality of life. The overall prevalence of IA was 23.7%, with 32.5% in Macau and 19.8% in mainland China. Students in Macau were more likely to suffer from IA than those in mainland China (OR = 2.15, p < 0.001). Correlates of IA included being in higher school grades, poor academic performance, and more severe depressive symptoms. Students with IA reported lower quality of life in physical, psychological, social, and environmental domains. IA is common among Chinese adolescents, particularly in Macau. Considering the negative impact of IA on health and quality of life, regular screening and effective interventions should be undertaken for young Internet users.

## Introduction

Internet addiction (IA) is a recently identified health problem, common among adolescent and young adult populations. IA usually refers to a persistent and recurrent maladaptive behavior, causing distress and significant functional impairments^1^. It is also named “problematic Internet use”, “pathological Internet use”, or “compulsive Internet use”. To date, there has been no agreement about the diagnostic criteria for IA. IA involves different types of online behaviors, such as online gaming, online shopping, online pornography, online gambling, and social media use. Due to its associations with a host of physical^2^, psychological^3,4^ and interpersonal problems^5^ among adolescents, IA has gained growing concerns in both research and clinical practice. For instance, "Internet gaming disorder" and the relevant proposed diagnosis criteria were described in the section recommending conditions for further research in the Diagnostic and Statistical Manual of Mental Disorders (DSM-5)^6^, and "gaming disorder" was classified as a psychiatric disorder in the International Classification of Diseases (ICD-11)^7^.

Some studies have examined the epidemiology of IA, but findings vary greatly across countries, due to different sampling methods, definitions and assessment instruments, and sociocultural contexts^8,9^. For instance, the prevalence of IA among adolescents ranged from 1.3% in Turkey^10^ to 12.1% in Italy^11^ using the Young’s Internet Addiction Test (IAT) , from 15.2% in Greece^12^ to 17–26.8% in Hong Kong using the Internet Addiction Diagnostic Questionnaire (IADQ)^13^, and 17.4% in Taiwan using the Chen’s Internet Addiction Scale (CIAS)^14^. The updated report from the Chinese Internet Network Information Center (CNNIC) revealed that 17.3% of Chinese Internet users aged < 18 years had Internet dependence^15^.

IA is often comorbid with psychiatric disorders and related problems^16,17^, such as attention deficit and hyperactivity disorder (ADHD)^18^, depression and anxiety^19,20^, aggressive behaviors^21^, substance use disorder (e.g., alcohol abuse) ^22^, and suicidal behaviors ^23,24^. Moreover, certain demographic factors are also associated with IA, such as obesity^25^, higher school grades^26^, poor academic performance and engagement in high-risk behaviors^27^, higher family income^28^, and lower level of parental attachment^29^. IA is significantly determined by sociocultural contexts^8^. Therefore, the epidemiology of IA should be examined in different sociocultural and economic contexts separately.

As a special administrative region, Macau is situated at the southeastern tip of China with independent legal and political systems. After four centuries of Portuguese administration, Macau was returned to China’s jurisdiction in 1999. Macau is a society greatly affected by both Eastern and Western cultural values, which is quite different from other areas of China in many socio-cultural and economic aspects. Following the opening policy of the casino industry in 2002, Macau experienced an economic boom, with its GDP per capita ranking top five globally ^30^. The rapid economic growth and social changes have a great impact on adolescents. For instance, many young people can find high paying jobs with low qualifications in the casino industry, therefore, some of them had inadequate motivation for academic achievement, dropped out of schools^31^, and even experienced mental health and behavioral problems, such as depression, smoking, alcohol drinking and illicit drug use^32^. All of these issues may increase the risk of IA among adolescents^33,34^. Therefore, understanding the epidemiology of IA and its associated factors is important for policymakers and health authorities to develop preventive measures and appropriate treatment to reduce the risk of IA and related health problems within this population.

A previous study found significantly higher IA prevalence among adolescents in Hong Kong (a city having similar historical and socio-cultural background with Macau) than that in mainland China^35^. To date, however, little is known about problematic Internet use among adolescents in Macau and no published studies compared the epidemiology of IA in Macau and mainland China. Importantly, these findings may have great implications for policymakers and health professionals in areas which experienced rapid economic growth and social changes. Therefore, we compared the prevalence of IA among adolescents between Macau and mainland China, along with key socio-demographic correlates of IA and quality of life (QOL) in both populations. We hypothesized that due to different sociocultural and economic contexts, IA prevalence would be significantly different among adolescents between Macau and mainland China. We also predicted that IA would be negatively associated with QOL.

## Methods

### Setting, subjects and data collection

This was a cross-sectional study that was carried out in Macau and three cities in mainland China (Beijing, Hefei and Shenzhen) from April to July 2017. Beijing, Hefei and Shenzhen are located in northern, central and southern China, which represents a range of major geographic settings in mainland China.

According to the number of students in each school, one secondary school was selected from Macau, Beijing and Hefei, while five secondary schools were selected from Shenzhen. Two to six classes with 120–200 students in each grade within participating schools were randomly selected according to their total number of students to participate in this study. Students who met the following criteria were included: (1) adolescents aged between 10 and 18 years; (2) Chinese ethnicity; (3) fluency in Chinese language (Cantonese or Mandarin). There was no exclusion criterion. The trained team members or teachers guided the students to complete the data collection forms in the classroom and then collected the forms. All the data were collected on a voluntary and anonymous basis. The research protocol was reviewed and approved by the Research Ethics Committee of the University of Macau. All methods were performed in accordance with the Declaration of Helsinki. Oral informed consent was obtained from students and written informed consent was signed by students’ parents or guardians.

### Assessment instruments and measures

A data collection form was designed for this study to collect socio-demographic information, such as age, gender, study site, religious belief, grade, self-perceived psychical health, academic performance and stress, and relationship with classmates, teachers and family. Socioeconomic data were also recorded, such as perception of family financial status, parental educational level, parental work status and marital status^28,36^. Parental educational level referred to the highest education level obtained by either the participant’s father or mother, with three levels, i.e., high level: completion of at least undergraduate studies, medium level: completion of secondary school studies, and low level: completion of primary school studies or not receiving any school education. Parental work status had two categories, i.e., unemployed: at least one parent was not employed, and employed: both parents were employed. Parental marital status had two categories, i.e., married: complete original family with both biological parents present, and other types: remarriage, divorce, separation, or one or both parents passed away.

The Young’s Internet Addiction Test (IAT)—Chinese version was used to assess the presence and severity of IA^1,37^. The IAT contains 20 items (from 1 = “rarely” to 5 = “always”), with the total score ranging from 20 to 100. The total score of > / = 50 points was considered as “having IA”^28,38^. The IAT had good psychometric properties in Chinese adolescents (e.g., Cronbach’s alpha was 0.90)^28,38^. The Center for Epidemiologic Studies Depression Scale (CESD)—Chinese version was used to assess the severity of depressive symptoms^39,40^. A higher score reflects more severe depressive symptoms. The Chinese version of World Health Organization Quality of Life-BREF (WHOQOL-BREF) was used to evaluate QOL^41,42^. WHOQOL-BREF is a 26-item self-reported scale to assess QOL in physical health, psychological health, social health and environmental domains.

### Statistical analysis

Data analyses were performed using SPSS V24.0. Comparisons of socio-demographic and clinical variables between Macau and mainland China, and between IA and non-IA groups were conducted using independent samples t-tests, Mann–Whitney U tests, and chi-square tests, as appropriate. Normal distributions of continuous variables were checked by one-sample Kolmogorov–Smirnov test. Binary logistic regression analysis with the “enter” method was used to examine IA prevalence between Macau and mainland China, with IA as the dependent variable, study site (Macau vs. mainland China) as the only independent variable, after controlling for variables that significantly differed in univariate analyses. In addition, analysis of covariance (ANCOVA) was used to compare QOL between IA and non-IA groups after controlling for variables that significantly differed in univariate analyses. Binary logistic regression analysis with “enter” method was used to examine the independent correlates of IA. IA was the dependent variable, while those with significant group differences in the above univariate analyses were entered as independent variables. Significance was set at 0.05, with two-tailed tests.

## Results

A total of 3380 students were invited to join this study, and 2892 agreed and completed the assessments: 882 in Macau, 500 in Beijing, 699 in Hefei, and 811 in Shenzhen. No significant differences in age and gender were observed between students who participated and those who refused to participate in this study. The basic demographic characteristics are shown in Table 1.

**Table 1:** Socio-demographic and clinical characteristics of Chinese adolescents in Macau and mainland China.

	Total sample (n = 2892)		Mainland China (n = 2010)		Macau (n = 882)		Statistics		
	N	%	N	%	N	%	Χ^2^	df^a^	p
Male	1527	53.8	1089	55.1	438	50.9	4.4	1	**0.04**
Religious beliefs	361	13.3	253	13.4	108	13.1	0.04	1	0.84
Only child	1072	40.3	900	48.4	172	21.5	168.1	1	** < 0.001**
Senior secondary school	1425	49.5	1074	53.7	351	39.9	46.6	1	** < 0.001**
Living with family	2303	80.1	1430	71.6	873	99.5	298.7	1	** < 0.001**
**Self-perceived physical health**							71.0	2	** < 0.001**
Good	1162	40.3	909	45.4	253	28.8			
Fair	1552	53.9	992	49.6	560	63.7			
Poor	167	5.8	101	5.0	66	7.5			
**Self-perceived weight**							15.7	2	** < 0.001**
Underweight	379	13.2	259	13.0	120	13.8			
Normal	1611	56.2	1084	54.2	527	60.7			
Overweight	878	30.6	657	32.9	221	25.5			
**Academic performance**							16.4	3	**0.001**
Excellent	574	20.3	422	21.5	152	17.6			
Good	878	31.0	632	32.2	246	28.4			
Fair	778	27.5	525	26.7	253	29.2			
Poor	600	21.2	385	19.6	215	24.8			
**Academic stress**							7.4	2	**0.02**
Little	299	10.4	226	11.2	73	8.3			
Fair	1874	64.9	1305	65.0	569	64.8			
Great	714	24.7	478	23.8	236	26.9			
**Relationships with classmates**							29.4	2	** < 0.001**
Good	1422	49.3	1053	52.5	369	42.1			
Fair	1358	47.1	894	44.5	464	52.9			
Poor	104	3.6	60	3.0	44	5.0			
**Relationships with teachers**							21.6	2	** < 0.001**
Good	980	34.1	735	36.7	245	28.0			
Fair	1789	62.2	1190	59.4	599	68.4			
Poor	109	3.8	77	3.8	32	3.7			
**Relationships with family**							63.4	2	** < 0.001**
Good	1956	68.0	1452	72.6	504	57.5			
Fair	851	29.6	506	25.3	345	39.4			
Poor	70	2.4	43	2.1	27	3.1			
**Perception of family income**							9.2	2	**0.01**
Affluent	346	12.0	255	12.8	91	10.4			
Enough	2391	83.1	1634	81.7	757	86.1			
Poor	141	4.9	110	5.5	31	3.5			
**Parental educational level**							22.5	2	** < 0.001**
High	768	26.6	585	29.1	183	20.7			
Medium	1959	67.7	1310	65.2	649	73.6			
Low	165	5.7	115	5.7	50	5.7			
**Parental work status**							24.3	1	** < 0.001**
Unemployed	656	23.2	510	25.7	146	17.2			
Employed	2175	76.8	1472	74.3	703	82.8			
**Parental marital status**							3.3	1	0.07
Married	2485	86.5	1747	87.3	738	84.7			
Others	388	13.5	255	12.7	133	15.3			
**IA**	684	23.7	397	19.8	287	32.5	55.5	1	** < 0.001**

	Mean	SD	Mean	SD	Mean	SD	Z	df^b^	P
Age	15.1	1.7	15.1	1.7	15.1	1.6	–0.02	-	0.98
CESD total score	16.1	9.8	15.5	9.9	17.6	9.4	–6.4	-	** < 0.001**
IA score	40.2	14.4	38.3	14.4	44.3	13.5	–11.7	-	** < 0.001**

Bolded values are p < 0.05; academic performance: excellent = 85–100, good = 75–85, fair = 65–75, poor =  < 65; Parental educational level: high = completion of at least undergraduate studies, medium = completion of secondary school studies, low = completion of primary school studies or not having completed any school study; Parental work status: unemployed = at least one parent is not employed, employed = both parents are employed; Parental marital status: married = complete original family with biological parents, others = remarriage, divorce, separation, or one or both parents passed away.

CESD: Center for Epidemiologic Studies Depression Scale; IA: Internet addiction.

^a^Chi-square test.

^b^Mann–Whitney U test.

The overall prevalence of IA was 23.7% (95% CI: 22.1–25.2%), with 19.8% (95% CI: 18.0–21.5%) in mainland China and 32.5% (95% CI: 29.4–35.6%) in Macau (x^2^ = 55.5, p < 0.001). There were significant differences between the Macau and mainland China in gender, being an only child, being senior secondary school students, living with family, self-perceived physical health and weight, academic performance and stress, relationships with classmates, teachers, and family, perception of family income status, parental educational level, parental work status and CESD score. No significant difference in IA prevalence between genders was found in mainland China (18.7% in boys vs. 20.5% in girls; x^2^ = 1.02, p = 0.3) and Macau (33.3% in boys vs. 32.2% in girls; x^2^ = 0.14, p = 0.71). After controlling for these variables, there were still significant differences between the two areas in IA prevalence (OR = 2.15, 95% CI: 1.68–2.74, p < 0.001).

The comparison of socio-demographic and clinical characteristics between the IA and non-IA groups is summarized in Table 2. Significant differences between the two groups were found in terms of age, study site, grade, self-perceived physical health and weight, academic performance and stress, relationships with classmates, teachers, and family, self-reported family income status, parental marital status and CESD score. After controlling for these variables, compared to the non-IA group, the IA group had a lower QOL in the physical (F = 4.5, p = 0.03), psychological (F = 6.2, p = 0.01), social (F = 7.1, p = 0.008) and environmental domains (F = 9.6, p = 0.002). Students in senior secondary school, with poor academic performance, and having more severe depressive symptoms were independently and significantly associated with higher risk of IA (Table 3).

**Table 2:** Socio-demographic and clinical characteristics of Chinese adolescents with and without IA.

	Non-IA (n = 2208)		IA (n = 684)		Statistics		
	N	%	N	%	Χ^2^	df^a^	p
Male	1177	54.3	350	52.4	0.7	1	0.39
School site (Macau)	595	26.9	287	42.0	55.5	1	** < 0.001**
Religious beliefs	269	13.0	92	14.4	0.9	1	0.35
Only child	832	40.9	240	38.2	1.5	1	0.22
Senior secondary school	1061	48.2	364	53.5	5.6	1	**0.02**
Living with family	1766	80.4	537	79.2	0.5	1	0.49
**Self-perceived physical health**					66.0	2	** < 0.001**
Good	965	43.9	197	28.9			
Fair	1137	51.7	415	60.9			
Poor	98	4.5	69	10.1			
**Self-perceived weight**					12.9	2	**0.002**
Underweight	281	12.8	98	14.5			
Normal	1272	58.0	339	50.2			
Overweight	640	29.2	238	35.3			
**Academic performance**					48.9	3	** < 0.001**
Excellent	485	22.4	89	13.4			
Good	701	32.3	177	26.7			
Fair	565	26.1	213	32.2			
Poor	417	19.2	183	27.6			
**Academic stress**					50.2	2	** < 0.001**
Little	231	10.5	68	10.0			
Fair	1497	67.9	377	55.2			
Great	476	21.6	238	34.8			
**Relationships with classmates**					54.6	2	** < 0.001**
Good	1153	52.4	269	39.4			
Fair	993	45.1	365	53.5			
Poor	56	2.5	48	7.0			
**Relationships with teachers**					37.4	2	** < 0.001**
Good	809	36.8	171	25.2			
Fair	1321	60.1	468	68.9			
Poor	69	3.1	40	5.9			
**Relationships with family**					89.9	2	** < 0.001**
Good	1588	72.2	368	54.3			
Fair	577	26.2	274	40.4			
Poor	34	1.5	36	5.3			
**Perception of family income**					6.5	2	**0.04**
Affluent	264	12.0	82	12.0			
Enough	1836	83.6	555	81.3			
Poor	95	4.3	46	6.7			
**Parental educational Level**					5.7	2	0.06
High	564	25.5	204	29.8			
Medium	1521	68.9	438	64.0			
Low	123	5.6	42	6.1			
**Parental work status**							
Unemployed	487	22.5	169	25.4	2.4	1	0.1
Employed	1678	77.5	497	74.6			
**Parental marital status**					12.6	1	** < 0.001**
Married	1927	87.8	558	82.4			
Others	269	12.2	119	17.6			

	Mean	SD	Mean	SD	Z	df^b^	P
Age	15.0	1.7	15.3	1.6	− 3.9	–	** < 0.001**
CESD total score	14.1	8.6	22.6	10.7	− 18.9	–	** < 0.001**

					F	df^c^	p
Physical QOL	14.6	2.5	13.1	2.4	4.5	–	**0.03**
Psychological QOL	13.6	3.1	12.1	3.0	6.2	–	**0.01**
Social QOL	14.5	3.6	13.4	3.7	7.1	–	**0.008**
Environmental QOL	13.5	3.1	12.7	2.8	9.6	–	**0.002**

Bolded values are p < 0.05; Academic performance: excellent = 85–100, good = 75–85, fair = 65–75, poor =  < 65; Parental educational level: high = completion of at least undergraduate studies, medium = completion of secondary school studies, low = completion of primary school studies or not having completed any school study; Parental work status: unemployed = at least one parent is not employed, employed = both parents are employed; Parental marital status: married = complete original family with biological parents, others = remarriage, divorce, separation, or one or both parents passed away.

CESD: Center for Epidemiologic Studies Depression Scale; IA: internet addiction; QOL: quality of life.

^a^Chi-square test.

^b^Mann–Whitney U test.

^c^Analysis of covariance.

**Table 3:** Socio-demographic and clinical characteristics independently associated with IA.

Variable	OR	95% CI lower limit	95% CI upper limit	p
Macau (ref: mainland China)	1.84	1.49	2.26	** < 0.001**
Senior secondary school^a^	1.24	1.01	1.51	**0.04**
CESD score	1.09	1.07	1.10	** < 0.001**
**Self-perceived physical health**				
Good	1	–	–	–
Fair	1.12	0.89	1.40	0.33
Poor	1.04	0.68	1.59	0.85
**Self-perceived weight**				
Normal	1	–	–	–
Underweight	1.03	0.77	1.38	0.84
Overweight	1.07	0.86	1.34	0.53
**Academic performance**				
Excellent	1	–	–	–
Good	1.24	0.91	1.69	0.17
Fair	1.63	1.20	2.22	**0.002**
Poor	1.73	1.26	2.39	** < 0.001**
**Academic stress**				
Little	1	–	–	–
Fair	0.79	0.56	1.12	0.19
Great	0.92	0.63	1.34	0.67
**Relationships with classmates**				
Good	1	–	–	–
Fair	0.81	0.63	1.02	0.08
Poor	0.89	0.52	1.53	0.68
**Relationships with teachers**				
Good	1	–	–	–
Fair	1.18	0.92	1.52	0.20
Poor	0.80	0.46	1.41	0.45
**Relationships with family**				
Good	1	–	–	–
Fair	1.13	0.91	1.40	0.28
Poor	1.09	0.61	1.95	0.76
**Perception of family income**				
Affluent	1	–		–
Enough	0.89	0.66	1.21	0.45
Poor	0.84	0.50	1.40	0.50
**Parental marital status**				
Married	1			-
Others	1.16	0.88	1.53	0.29

Bolded values are p < 0.05; academic performance: excellent = 85–100, good = 75–85, fair = 65–75, poor =  < 65; parental marital status: married = complete original family with biological parents, others = remarriage, divorce, separation, or one or both parents passed away.

CESD: Center for Epidemiologic Studies Depression Scale.

^a^The variable “age” and “senior secondary school” showed collinearity, only “senior secondary school” was included in the logistic regression.

## Discussion

To the best of our knowledge, this was the first study to compare the prevalence of IA between adolescents in Macau and those in mainland China. IA is common in Chinese adolescents, particularly in Macau. Using the same IAT cutoff value, our findings were relatively higher than the figures reported in Western countries and other areas, for example, 5.8–12.1% in Italy^11,43^, 11.7% in Switzerland^44^ and 7.9–18.2% in Turkey^10,45^. The prevalence in mainland China in this study was also higher than the corresponding figures among Chinese adolescents in previous studies, for example, 8.1% in eight cities in China^28^, 10.4% in Anhui province^46^ and 12.2% in Guangdong province using the same IAT cutoff value^47^. The rapid growth of Internet use in China in recent years may contribute to the increased prevalence of IA in this study. According to the report from the CNNIC, the number of Internet users aged < 18 years was around 175 million in China, which accounted for 93.1% of this population^15^, with an annual growth rate of 3.7—8.1%^48^. In addition, widespread use of smartphones makes access to the Internet much easier than before. It was reported that 93.9% of Chinese adolescents accessed the Internet using their smartphones^15^. Furthermore, a rapid increase of online learning, entertainment and leisure activities made adolescents spend more time using the Internet^15^. All these factors are associated with increased prevalence of IA in China. It should be noted that no gender difference in IA prevalence was found in this study, indicating IA commonly occurs in both genders^49,50^.

Students in Macau had a significantly higher IA prevalence than those in mainland China, which is similar to previous findings comparing IA prevalence between mainland China (17.1%) and Hong Kong (31.6%) using the same instrument^35^. IA is more prevalent in Macau than that in mainland China. Based on previous findings that IA was associated with high family income and socioeconomic status^28^, we speculated that higher IA prevalence in Macau is most likely due to economic and sociocultural reasons. Macau's per capita GDP ranks top five globally^30^, almost nine times higher than mainland China. Thus, with higher household income, adolescents in Macau may have access to electronic devices at an earlier age. A study found that 51.1% of adolescents had one personal computer in Hong Kong, while the corresponding figure was only 14.7% in mainland China^35^. This situation may be more widespread in Macau since the economic conditions in Macau are better still than in Hong Kong. Moreover, the wireless network infrastructures are well built in Macau, providing easy and free access to the Internet, and additional features (e.g., Facebook, and YouTube) of the internet are available in Macau, while these platforms are not widely available in mainland China. Additionally, as a former European colony, Macau is greatly affected by both Eastern and Western cultures, which may lead to different parenting styles regarding Internet use between Macau and mainland China^51^. Due to high degree of urbanization and good economic status, adolescents in Macau usually do not experience job-seeking pressures following graduation. Moreover, the living cost has rapidly increased in Macau in recent years, so adolescents’ parents typically work, which results in decreased time to take care of their children. In contrast, their counterparts in mainland China face fierce competition in school, and they are highly occupied with homework, and therefore have limited time online with more strict parental monitoring^15,52^. All these factors likely contribute to the higher IA prevalence in Macau.

Higher school grades, poor academic performance and depressive symptoms were significantly associated with IA. Previous studies found that older adolescents were more likely to be addicted to the Internet^26,28^, which could be explained by several reasons. Compared to younger adolescents, older adolescents usually experience a more rapid phase of psychosocial, cognitive and intellectual development, and they start to take certain adult roles and consider long-term career goals. Thus, they often need to use Internet for information and social activities. Moreover, students in higher school grade are easier to access to the Internet^26,28^. Therefore, older adolescents are more likely to report higher IA rate. It may also be true that older adolescents are using their devices for online gaming, which is known to be highly addictive and increase in use around this age^53,54^.

The association between IA and poor academic performance found in this study supported earlier findings^27,55^. Studies found that “loss of control” moderates the association between IA and declining school performance^55^. Students suffering from IA usually spend a lot of time online, regardless of their school responsibilities. Some of them even skip class and sacrifice sleep time, resulting in fatigue and low degree of involvement in classes. The stress under the poor school performance may lead to further involvement of online activities for adolescents in order to escape negative emotions^56^.

Similar to previous studies^46,57^, we found the positive association between depressive symptoms and IA. Previous studies found that persons with IA usually lose rewarding experiences in their offline world and then feel socially isolated, which could increase the risk of depressive mood^20,58^; in addition, those with depressive symptoms often use Internet to “self-medicate” as Internet use could alleviate depressive emotion^4,59^. The internet constructs a virtual and unrealistic world in which those with depression may obtain social support, self-identity, and the sense of achievement that cannot be satisfied in real world^16^. Nevertheless, excessive Internet use may impair daily functions and further worsen dysregulated mood. Some studies found a bidirectional association between depression and IA^60^.

Long-term Internet use and IA may cause physical problems, such as reduced physical activities and obesity^61^, physical pain (e.g., neck, shoulder and back pain)^62^, hearing impairment and impaired vision^63^, increased risk of accidents (e.g., falling, slipping, and bumps/collisions)^64^, disturbed circadian rhythm^65^, and poor sleep quality^2,65^. In addition, persons suffering from IA often have emotional and social problems, which may isolate themselves from peers, make them spend more time online and finally lead to additional adaption stress, and psychological and interpersonal relationship problems^3^. All these factors could cause lower QOL. As expected, IA was negatively associated with all QOL domains in this study, which is consisted with the findings of a meta-analysis^66^.

The strengths of this study included the large sample size, multisite design, random sampling, and the use of standardized instruments. Some limitations need to be noted. First, this was a cross-sectional study, therefore, the causal relationships between IA and a host of demographic and clinical variables are tentative. Second, due to logistical reasons, only three cities in mainland China were selected to represent the adolescents in central, eastern, and southern China. Therefore, the findings cannot be generalized to all parts of China, particularly rural areas. Third, the possibility of recall bias cannot be excluded. Fourth, the IAT only measures IA as a general construct, therefore, subtypes of IA were not measured in this study. In addition, there have been no standardized instruments on socioeconomic and cultural factors in China. Instead, a host of categorical variables, such as perception of family status, parental educational level, parental work status and marital status, were recorded as the index of socioeconomic status. Finally, some potentially confounding factors were not examined in this study, such as physical diseases and physical exercise.

In conclusion, IA is common in Chinese adolescents, especially in Macau. Given the negative impact of IA on health outcomes and quality of life, more attention should be paid to young Internet users, and regular screening and effective interventions should be undertaken for this population.

## Data Availability

The Research Ethics Committees of the University of Macau that approved the study prohibits the authors from making the research data set publicly available. Readers and all interested researchers may contact Prof. Yu-Tao Xiang (Email address: xyutly@gmail.com) for details. Prof. Xiang could apply to the Research Ethics Committees of the University of Macau for the release of the data.
